# Revised network loadings

**DOI:** 10.3758/s13428-025-02640-3

**Published:** 2025-03-14

**Authors:** Alexander P. Christensen, Hudson Golino, Francisco J. Abad, Luis Eduardo Garrido

**Affiliations:** 1 Department of Psychology and Human Development, Vanderbilt University, Nashville, TN 37203 USA; 2 University of Virginia, Charlottesville, VA USA; 3 Universidad Autónoma de Madrid, Madrid, Spain; 4 Pontificia Universidad Católica Madre y Maestra, Santiago de los Caballeros, Dominican Republic

**Keywords:** Loadings, Network analysis, Psychometrics

## Abstract

Psychometric assessment is the foundation of psychological research, where the accuracy of outcomes and their interpretations depend on measurement. Due to the widespread application of factor models, factor loadings are fundamental to modern psychometric assessment. Recent advances in network psychometrics introduced network loadings which aim to provide network models with a metric similar to factor loadings to assess measurement quality when the data are generated from a factor model. Our study revisits and refines the original network loadings to account for properties of (regularized) partial correlation networks, such as the reduction of partial correlation size as the number of variables increase, that were not considered previously. Using a simulation study, the revised network loadings demonstrated greater congruence with the simulated factor loadings across conditions relative to the original formulation. The simulation also evaluated how well correlations between factors can be captured by scores estimated with network loadings. The results show that not only can these network scores adequately estimate the simulated correlations between factors, they can do so without the need for rotation, a standard requirement for factor loadings. The consequence is that researchers do not need to choose a rotation with the revised network loadings, reducing the analytic degrees of freedom and eliminating this common source of variability in factor analysis. We discuss the interpretation of network loadings when data are believed to be generated from a network model and how they may fit into a network theory of measurement.

**Supplementary Information:**

The online version contains supplementary material available at 10.3758/s13428-025-02640-3.

Psychometric assessment is at the foundation of psychological research. Measurement of psychological attributes can affect the extent to which the results, and subsequent conclusions, are valid. Latent variable models, especially factor models, have dominated psychological measurement over the last century (Borsboom et al., 2003). A factor model assumes that a latent variable or set of latent variables underlie the relationships between observable variables. The extent to which an observable variable measures a latent variable is indicated by its factor loading. Factor loadings are frequently used to assess an observable variable’s measurement quality (DeVellis, 2017).

Over the last decade, network models have emerged as an alternative psychometric method termed *network psychometrics* (Epskamp & Fried, 2018). Psychometric networks represent observable variables as nodes (circles) and relationships between them, such as partial correlations, as edges (lines). The estimation and representation of a network does not explicitly assume that latent variables underlie the relationships between the observable variables (Borsboom, 2017; Guttman, 1953). Instead, these models suggest that relationships between observable variables occur *directly*, mutually reinforcing one another (known as mutualism; van der Maas et al., 2006).

Centrality measures, which quantify the relative position of a node relative to other nodes in the network, are commonly used as measurement metrics of observable variables in psychometric networks (Bringmann et al., 2019). Recent research has linked some centrality measures such as *node strength* or the absolute sum of a node’s connections to other nodes in the network to confirmatory factor analysis (CFA) loadings (Hallquist et al., 2021). A key finding from this work is that node strength represents a composite of latent causes that were simulated in the data. This result demonstrates that although latent variables are not explicitly estimated by network models, centrality measures are affected by their presence. This consequence led to the development of so-called *network loadings* (Christensen et al., 2021) that represent each node’s strength split by *communities* or sets of densely connected nodes in the network that are consistent with latent factors when data are generated from a factor model (Christensen et al., 2024, 2020; Golino & Epskamp, 2017; Golino et al., 2020).

Because network models do not estimate latent factors, the communities and loadings derived from networks represent heuristic measures of latent factors and their loadings, respectively, when the data are generated by a factor model. These heuristics are useful to understand how factor and network models can be connected but also provide an analogous statistic when the data may not be generated from a factor model. When data are generated from a network model, the communities represent emergent summaries of the mutual interactions between the constituent variables (Cramer, 2012) and the loadings represent the extent to which each node contributes to the emergence of a coherent community (Christensen et al., 2020; Ouyang et al., 2023; van Bork et al., 2019).

The goal of this study is to revisit and refine Christensen and Golino’s (2021) formulation of network loadings. We begin by introducing the original formulation of network loadings. Next, we identify conditions that were not included in the original simulation where the original network loadings become less congruent with the simulated factor loadings. These conditions are then used to motivate a revised formulation of network loadings. After, a simulation study is performed to demonstrate the improvement of the revised formulation over the original when data are generated from a factor model. These improvements pave the way for network loadings to provide more precise psychometric information. In addition, we evaluate whether rotations are necessary for network loadings, finding that, unlike factor loadings, rotations are unnecessary to arrive at an interpretable loading matrix and accurate correlations between factors.^1^

## Original network loadings

The procedure to estimate network loadings provided by Christensen and Golino (2021) starts by first performing Exploratory Graph Analysis (EGA; Golino & Epskamp, 2017; Golino et al., 2020). EGA begins by estimating a network, $\textbf{W}$, using the graphical least absolute shrinkage and selection operator (GLASSO; Friedman et al., 2008) with extended Bayesian information criterion (EBIC; Chen & Chen, 2008) model selection (EBICglasso; Epskamp & Fried, 2018). This network represents variables as nodes and regularized partial correlation as edges. On this network, a community detection algorithm, such as the Walktrap algorithm (Pons & Latapy, 2006), is applied to estimate the number and content of the communities in the network. The network and communities are then used to estimate the network loadings. The formal definition starts by defining node strength,

$$ s_i = \sum _{j=1}^p | w_{ij} |, $$

where $p$ is number of nodes in the network and $w_{ij}$ is the *edge weight* or (regularized) partial correlation between node $i$ and $j$. To derive a node’s strength for each community, these edge weights are split between each community, $c$,

1 $$\begin{aligned} l_{ic} = \sum _{j \in c}^p | w_{ij} |, \end{aligned}$$

where $l_{ic}$ is the unstandardized network loading for node $i$ in community $c$. To standardize the network loadings, the following formula is used,

2 $$\begin{aligned} o_{ic} = \frac{l_{ic}}{\sqrt{\sum _{i}^p l_{ic}}}, \end{aligned}$$

where $o_{ic}$ is the standardized network loading for node $i$ in community $c$. The resulting loading matrix, $\textbf{O}$, represents the *absolute* standardized network loadings.

**Fig. 1:**
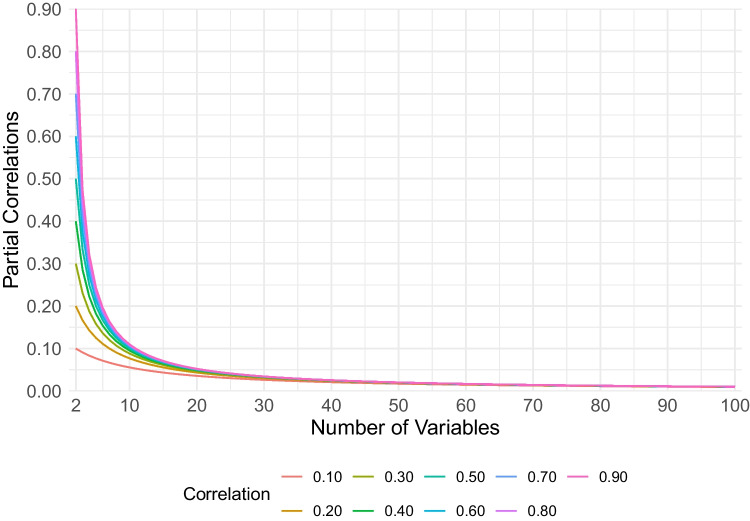
Partial correlations by the number of variables for a given zero-order correlation matrix of all one correlation value

Signs are added in a two-stage procedure. In the first stage, a “target” network, $\textbf{T}_w = w_{i \in c j \in c}$, or a symmetric matrix representing a sub-network of $\textbf{W}$ such that the rows and columns represent the nodes in a single community, $c$, and the elements represent edge weights between them. From this target network, the signs of each weight are obtained forming a matrix of signs, $\textbf{M}_w$. The sums of the signs, $\Sigma {\textbf {M}}_w$, is obtained. Signs are then determined by sequentially looping over each node in $\textbf{T}_w$ and determining whether flipping its sign increases $\Sigma {\textbf {M}}_w$. If there is an increase, then the sign for the node in the community is flipped, $o_{i \in c c} = -o_{i \in c c}$; otherwise, the sign stays the same. The goal of this procedure is to flip (partial) correlations to align in a positive direction for each community. This procedure is completed for each community before moving on to the second stage.

In the second stage, a “target” network, $\textbf{T}_b = w_{i \in c j \in k}$, where $k \ne c$, is created. $\textbf{T}_b$ is an asymmetric matrix representing a sub-network of $\textbf{W}$ such that the rows represent nodes in community $c$, columns represent nodes in community $k$, and elements represent the weights between them. The same procedure as the first stage is then followed such that a matrix, $\textbf{M}_b$, representing the sign of each weight in $\textbf{T}_b$ is created. The sums of the signs, $\Sigma {\textbf {M}}_b$, is obtained. Similar to the first stage, signs are determined by sequentially looping over each node in $\textbf{T}_b$ and flipping signs for $o_{i \in c j \in k}$ when there is an increase in $\Sigma {\textbf {M}}_b$.

After this two-stage procedure, all loadings in each community, $c$, are flipped to maintain an orientation toward greater positive loadings than negative loadings such that,

$$ o_{:c} = \left\{ \begin{array}{c l} o_{:c}, & \text {if} \ \sum _{i=1}^n o_{ic} > \sum _{i=1}^n -o_{ic} \\ -o_{:c}, & \text {otherwise} \end{array} \right. . $$

where $:$ represents all values in the row (first subscript) or column (second subscript). The result is a signed standardized network loading matrix, $\textbf{O}$. This formulation of network loadings will be referred to as the *original network loadings* hereafter. When network loadings are based on partial correlation networks, which are the most common in psychological literature (Borsboom et al., 2021), the loading sizes tend to be smaller in scale relative to factor loadings (e.g., Table 1, Table 2, Figure 1).

## Original simulation

The original network loadings were evaluated in a simulation study that compared their association with simulated factor loadings against CFA and EFA loadings (Christensen et al., 2021). The design of the simulation allowed loadings on each factor to vary between 0.40 and 0.70. The cross-loadings were randomly drawn from a normal distribution with a mean of zero and standard deviation (0.050, 0.075, 0.100, 0.125) that increased with the corresponding correlations between factors (0.00, 0.30, 0.50, 0.70). Two, three, and four factors with four, eight, and 12 variables per factor were generated for sample sizes of 250, 500, 500, and 1000. The results of the simulation demonstrated that the original network loadings were more strongly related to the simulated loadings than CFA but were less related to them than EFA. Overall, EFA loadings were most congruent with the factor loadings generated in the simulation.

Although the simulation design covered a range of conditions encountered in applied research, there were several conditions that were not included that could impact the congruence of the original network loadings with the simulated factor loadings. For example, each variable’s loading on its dominant factor (largest loading) was positively signed. Negative signs were generated on the cross-loadings, but there was no direct investigation on whether the sign adding procedure formulated above was appropriate.

Another impact is inherent in the sum of the assigned network loadings relative to cross-loadings. Partial correlation networks have a zero on the diagonal of their matrix. The consequence of this zero-valued self-connection is that the assigned loadings have one less possible non-zero value to include in their sums than the sums for the cross-loadings. We demonstrate this consequence with an example: Assume factor 1 and 2 have six variables each and the variables are correctly assigned to each factor. When summing the assigned loadings for factor 1, a variable’s connection to itself is zero and therefore the total number of possible non-zero values to include in the sum is 5. When summing the cross-loadings from the variables in factor 2 to factor 1, the total number of possible non-zero values to include in the sum is 6.

Although many community detection algorithms aim to maximize modularity (i.e., maximize the number of connections within a community and minimizes the number of connections to other communities; Newman, 2006), it’s possible that a variable could be fully connected to a community that it does not belong to and consequently have more non-zero values included in its sum than is possible for a variable that belongs to the community even though there are the same number of variables in both communities. As a consequence of zero-valued self-connections, the assigned loadings are always short of the possibility of one non-zero value relative to their respective cross-loadings.

A third impact is the number of variables per factor. Partial correlations can be affected by the number of variables. Under common-factor theory, partial correlations tend to zero ($r_{xy|z} \rightarrow 0$), as the number of variables go to infinity ($p \rightarrow \infty$; Guttman, 1953). A consequence for the original network loadings is that as the number of variables per factors increase (as well as number of factors), the original network loadings will shrink. When common factors do not exist, such as under network theory (Borsboom, 2017), partial correlations can instead reverse signs or get stronger as the number of variables increase (van Bork et al., 2019).

In conditions where there are unequal number of variables per factor, the original network loadings would have a reduced size on the factors with more variables relative to an equivalent loading size on factors with fewer variables. In general, the more variables in the dataset, the smaller the network loadings will become making the reported effect size guidelines of small (0.15), moderate (0.25), and large (0.35) less consistent and reliable. Taken together, these impacts can lead to network loadings that are less congruent with factor loadings and potentially jeopardizes their validity in certain conditions.

## Revised network loadings

Motivated by these limitations, we derived a revised formulation of network loadings. The first step to compute the unstandardized network loadings is to determine signs of each variable by creating a “target” network, $\textbf{T} = w_{i \in c j \in c}$, or a symmetric matrix representing a sub-network of $\textbf{W}$ such that the rows and columns represent the nodes in a single community, $c$, and the elements represent edge weights between them. Signs for each variable are obtained by iteratively flipping the signs of nodes where the sum of their connections in $\textbf{T}$ are negative. These sign changes are tracked in a vector $\textbf{v}$ until all signs $\textbf{T}$ are in a predominantly positive orientation. The end result is a vector of signs for each node, $\textbf{v}$. The sum of the vector of signs, $\sum _{i \in c}^p v_i$, is used to determine whether the community should be recoded toward a positive orientation such that a negative value (i.e., $(\sum _{i \in c}^p v_i) < 0$) reverses all signs ($\textbf{v} = -\textbf{v}$).

The next step computes the assigned or “within” community loadings using the target network, $\textbf{T}$. For node $i$ in community $c$, the unstandardized network loading is denoted as $l_{ic}$ and computed as follows,

3 $$\begin{aligned} \text {within} \ l_{ic} = p_c \left( \frac{\sum _{j=1}^{p_c} t_{ij}}{p_c - 1} \right) , \end{aligned}$$

where $p_c$ is the number of nodes in community $c$ and $t_{ij}$ represents the weight of node $i$ and $j$ within the community. In contrast to Eq. 1, this formulation takes into account that a node’s connection with itself is equal to zero in $\textbf{W}$. The sum of a node’s connections within its community is divided by the number of nodes in the community minus itself ($p_c - 1$), resulting in an adjusted average weight for node $i$ in community $c$. This adjusted average weight is then multiplied by the number of nodes ($p_c$) in the community providing an adjusted sum for node $i$. This adjustment ensures that when the same nodes are used in the sum for the cross-loadings (i.e., between-community sum) they will reflect the same number of nodes used in the within-community sum. This adjustment reduces issues related to cross-loadings being larger than the loadings in the assigned community due to the potential of an additional node being used in their computation.

Turning to the cross-loading formulation, the unstandardized cross-loading or “between” community loading starts by flipping the signs in the original network, $\textbf{W}$, based on the vector of signs, $\textbf{v}$, from the first step. Then, the cross-loadings are computed,

$$ \text {between} \ l_{ik} = \sum _{j=1}^{p_k} w_{i \in c j \in k}, $$

where $p_k$ is the number of nodes in community $k$. The result of these formula for the within- and between-community loadings is the unstandardized network loading matrix, $\textbf{L}$. Each row (node) of $l_{i:}$ is then multiplied by its corresponding signs, $v_i$, to ensure that the proper signs are applied to the loading matrix, consistent with the original variable orientation.

Finally, the third revision to the original network loadings is their standardization. As mentioned before, as the number of variables increases toward infinity ($p \rightarrow \infty$), the partial correlations tend to zero ($r_{x,y|z} \rightarrow 0$; Guttman, 1953) under certain circumstances (van Bork et al., 2019). This fact can be demonstrated by generating correlation matrices that have the same zero-order correlation value and computing the partial correlations as the number of variables increase (Fig. 1). The relationship between the partial correlations and number of variables, when the zero-order correlation for all variables is equal, roughly follows the form, $\frac{1}{\log {(p)}}$.^2^

Based on this consequence of partial correlations, an adjustment for the number of variables per factor is necessary. In the original standardization (Eq. 2), the sum across all unstandardized loadings for each factor is obtained and the square root is taken. This calculation has an implicit assumption that the sum will be proportionate to the number of variables per factor and therefore standardize the smaller loadings (due to more variables per factor) with a relatively smaller sum. In Christensen and Golino’s (2021) original simulation, the number of variables per factor were always equal on all factors (4, 8, or 12), concealing the consequences of their original standardization procedure.

To account for the number of variables per factor in the revised standardization, two adjustments were made. First, the sums of the assigned unstandardized loadings (Eq. 3) are used in the denominator rather than the sums across the unstandardized loadings for each factor. This adjustment is based on the notion that the covariance that is conditioned out of the relationship between any two partial correlations is due mainly to the common covariance shared by variables on the same factor. Second, based on the tendency of partial correlations to go to zero as the number of variables increase to infinity (Fig. 1), the form of $\frac{1}{\log {(p_c)}}$ is used to adjust for the number of variables per factor. Importantly, these adjustments assume that the underlying data generating model is a factor model and need further validation with other data generating mechanisms. These two adjustments lead to the revised standardization,

4 $$\begin{aligned} o_{ic} = \frac{l_{ic}}{\root \log (\zeta p_c) \of {\sum | \text {within} \ l_{:c}} |}, \end{aligned}$$

where $\zeta$ is a scaling factor for the magnitude of the loadings and $\zeta = 2$ by default.

## Comparison with original loadings

Three simulated data examples were generated to demonstrate how these revisions mitigate the limitations of the original network loadings. For the first example, a two factor model with six variables per factor and negative signs on the first three variables of each factor was used to generate data. The loadings for the variables on each factor were generated from a uniform distribution ranging from 0.45–0.65. There were no cross-loadings or correlations between factors and the sample size was 100,000. Table 1 shows the simulated loadings, original network loadings, and revised network loadings with the target variables and their signs highlighted. From the table, it is clear that the original network loadings do not have the appropriate signs whereas the revised network loadings do.

**Table 1:** Signed loading example

	Simulated		Original		Revised	
	1	2	1	2	1	2
V01	-0.63	0.00	-0.37	0.00	-0.48	0.00
V02	-0.64	0.00	-0.38	0.00	-0.49	0.00
V03	-0.51	0.00	-0.27	0.00	-0.35	0.00
V04	0.62	0.00	-0.36	0.00	0.46	0.00
V05	0.58	0.00	-0.33	0.00	0.42	0.00
V06	0.55	0.00	-0.31	0.00	0.39	0.00
V07	0.00	-0.60	0.00	0.36	0.00	-0.45
V08	0.00	-0.48	0.00	0.26	0.00	-0.34
V09	0.00	-0.58	0.00	0.34	0.00	-0.44
V10	0.00	0.59	0.00	0.35	0.00	0.45
V11	0.00	0.54	0.00	0.31	0.00	0.40
V12	0.00	0.59	0.00	0.35	0.00	0.45

For the second example, another simulated two factor example was generated, this time with three variables on the first factor and nine variables on the second factor. The loadings for the variables on each factor were generated from a uniform distribution ranging from 0.45–0.65 and there were no cross-loadings except on variable V04. This variable had equivalent loadings of 0.40 on the first and second factors. There was a moderate correlation between the factors (0.30) and the sample size was 100,000.

**Table 2:** Cross-loading example

	Simulated		Original		Revised	
	1	2	1	2	1	2
V01	0.63	0.00	0.34	0.06	0.46	0.08
V02	0.64	0.00	0.34	0.06	0.47	0.08
V03	0.51	0.00	0.27	0.04	0.37	0.05
**V04**	**0.40**	**0.40**	**0.34**	**0.24**	**0.32**	**0.35**
V05	0.00	0.58	0.00	0.29	0.00	0.44
V06	0.00	0.55	0.00	0.27	0.00	0.41
V07	0.00	0.60	0.00	0.31	0.00	0.46
V08	0.00	0.48	0.00	0.22	0.00	0.33
V09	0.00	0.58	0.00	0.29	0.00	0.44
V10	0.00	0.59	0.00	0.30	0.00	0.45
V11	0.00	0.54	0.00	0.26	0.00	0.40
V12	0.00	0.59	0.00	0.31	0.00	0.46

**Fig. 2:**
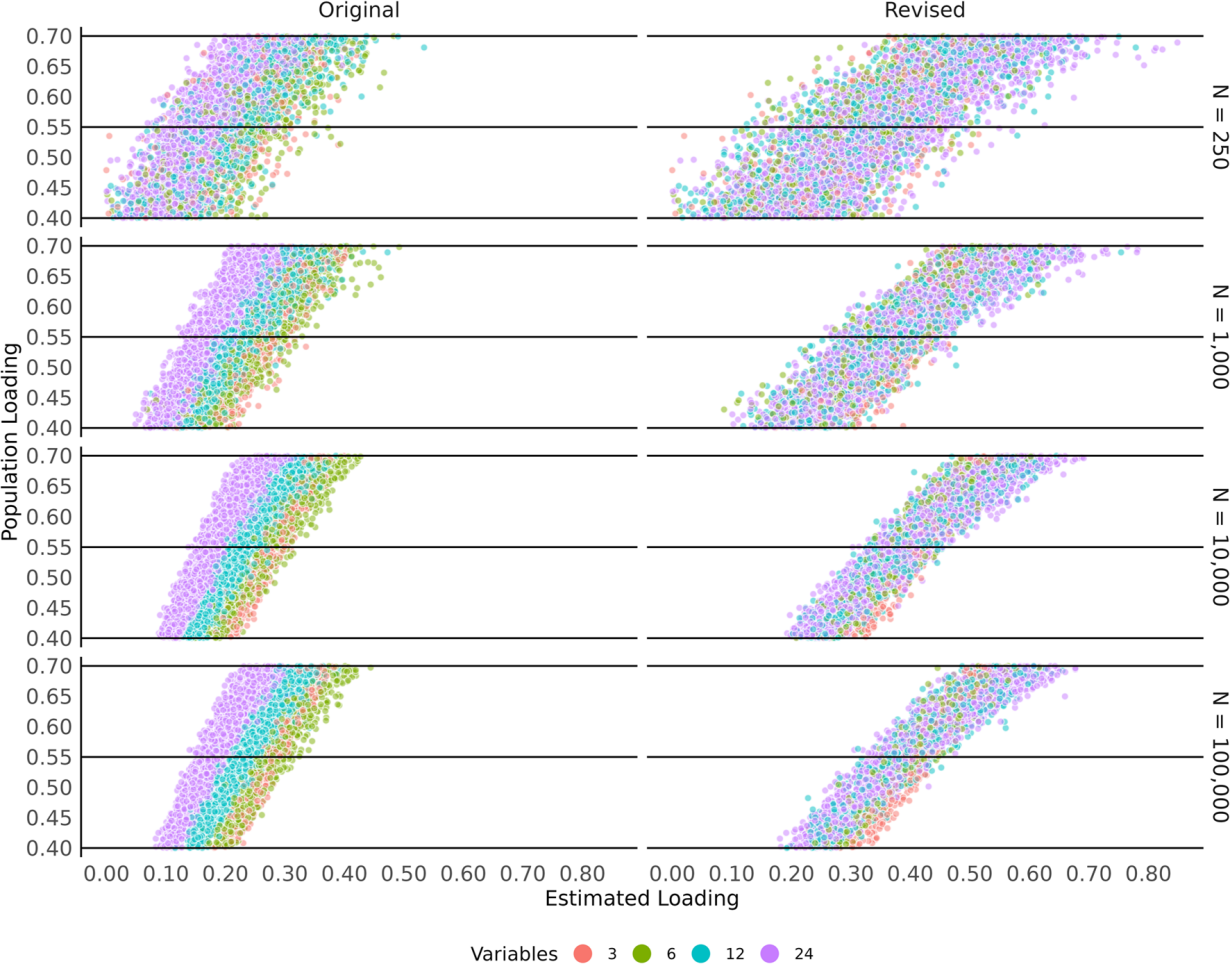
Correspondence between simulated factor loadings given 3, 6, 12, and 24 variables per factor and the original and revised network loadings. Both the original and revised network loadings are on different scales from each other as well as the factor loadings

From Table 2, the original network loadings appear to favor V04 on the first factor (0.34) relative to the second factor (0.24). In an applied setting, a researcher might make the distinction that V04 belongs to the first factor with a large cross-loading on the second factor (rather than an even split). Further, the loading on the first factor for V04 is as large or larger than the other assigned loadings on the first factor (V01 = 0.34, V02 = 0.34, V03 = 0.27) despite the simulated loadings being much larger (V01 = 0.63, V02 = 0.64, V03 = 0.51) than the target loading (0.40). For the revised network loadings, this issue is largely mitigated with the loadings on the first and second factor being roughly equivalent in size.

For the third example, a small simulation was performed. In this simulation, data were generated from a four-factor model with 3, 6, 12, and 24 variables on each factor, respectively. Loadings were generated from a uniform distribution between 0.40 and 0.70 and cross-loadings were generated from a normal distribution with a mean of zero and standard deviation of 0.05. Correlations between factors was constant at 0.30 and sample sizes of 250, 1000, 10,000, and 100,000 were generated. One hundred samples were generated to allow for variability in the loading structures. The aim of this small simulation is to demonstrate the dependence of the original network loadings on the number of variables per factor and how the revised standardization breaks this relationship.

Figure 2 displays the results of the small simulation broken down by sample size, ignoring the cross-loadings and focusing only on the assigned loadings. For both the original and revised network loadings, there is a clear linear pattern with the simulated (population) loadings. The original network loadings, however, show a pattern where as the number of variables increase, the loading size decreases. This dependence on the number of variables per factor for the original network loadings is clear in the Pearson’s correlation between them, $r = -0.49$. The revised network loadings do not show this pattern and have a negligible correlation with the number of variables per factor, $r = 0.02$. Further, the revised network loadings show a much stronger correlation overall with the simulated loadings ($r = 0.87$) relative to the original network loadings ($r = 0.74$).

Taken together, the revised network loadings make several improvements over the original network loadings: signs are consistent, community detected assigned loadings are larger than cross-loadings, and negligible dependence on number of variables per factor. These improvements are expected to lead the revised network loadings to be more congruent with factor loadings when the data are generated from a factor model. To evaluate this claim, we performed a simulation study that expands on Christensen and Golino’s (2021) work.

## Present research

The goal of the simulation study is to evaluate the revised network loadings under a variety of conditions that include conditions where the original network loadings break down. The simulation design followed closely to Christensen and Golino’s (2021) second simulation but added several important conditions. The first addition includes adding negative loadings to half the variables on each factor. As demonstrated above, this condition is intended to demonstrate the (in)appropriateness of the sign procedure of the original and revised network loadings. The second addition includes conditions where number of variables per factor (3–12), loadings (0.40–0.80), and correlations between factors (0.00–0.70) are randomly varying. The third condition, which was not considered in Christensen and Golino’s (2021) simulations, is whether a loading rotation can improve the recovery of the population loadings for the network loadings.

**Fig. 3:**
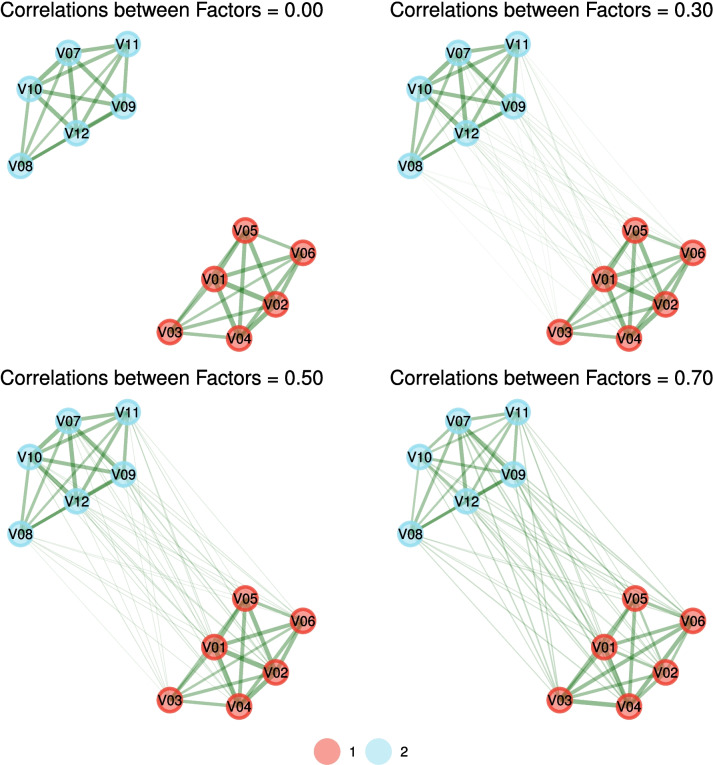
Four network models with the same loadings matrix but increasing correlations between factors

Loading rotations are numerous and their role in factor analysis is essential (Sass & Schmitt, 2010). Rotations aim to simplify the loading matrix structure to increase interpretability. After a rotation is applied, the loading matrix approaches a simple structure relative to the unrotated matrix. A particular benefit of oblique rotation is the estimation of correlations between factors. These correlations between factors are important for theoretical interpretation but also for computing scores and additional modeling such as bifactor or hierarchical factor models (Jiménez et al., 2023). With network loadings, it is unknown whether rotations are necessary to obtain correlations between factors; however, our intuition is that they are not because the topology of a network “embeds” the correlation information between factors due to the regularized partial correlations (Fig. 3).

If the factors are orthogonal, then the communities in the network appear isolated and detached from one another—that is, there are no connections between communities (Golino & Epskamp, 2017). If there are correlations between factors, then the covariance between factors remains after conditioning, leading to connections between communities. As correlations between factors increase, the connections between communities uniformly increase (Fig. 3). In network loadings, these relatively uniform between-community connections are represented as cross-loadings that are roughly the same size for all variables on the correlated factors. If cross-loadings exist beyond the correlations between factors, then they are represented as larger cross-loadings over and above the average cross-loading.

The main expectation for the simulation study was that the revised network loadings would correspond to the simulated population loadings as well as or better than the original network loadings. An additional comparison of EFA loadings with geominQ rotation were added to provide a factor analytic benchmark. In addition to the congruence of network and factor loadings, the accurate estimation of the correlations between factors were evaluated. The expectation for the revised network loadings was that although rotation may benefit their congruence with factor loadings and recovery of the correlations between factors, the improvements would be marginal. Such a result would suggest that the revised network loadings can be used without rotation, eliminating a researcher degree of freedom.

## Methods

### Simulation design

The conditions simulated in this study were intended to represent common instances of real-world data that adhere to latent factor models (Comrey & Lee, 2013). All data generated in this study were continuous data without skew to mitigate confounding effects that were not relevant to assess the improvement of the revised network loadings over the original network loadings.

Data were generated with two, three, and four factors with 3, 6, and 12 variables per factor. Another number of variables per factor condition drew the number of variables on each factor randomly from a uniform distribution ranging from 3 to 12. The loadings on these factors were manipulated to be drawn randomly from a uniform distribution that were $\pm 0.10$ the set values of 0.40, 0.55, and 0.70. Another loading condition drew loadings randomly from a uniform distribution ranging from 0.30 to 0.80. There were two conditions for loading signs such that one condition had “all positive” signs and the other had half the number of variables on each factor with negative signs (for three variables per factor, only one variable had a negative sign), which is referred to as “half negative” hereafter. The cross-loadings were drawn randomly from a normal distribution with a mean of zero and standard deviation of 0.05. The correlations between factors were manipulated to be 0.00 (orthogonal), moderate (0.30), large (0.50), and very large (0.70). Another correlation between factors condition drew correlations randomly from a uniform distribution ranging from 0.00 to 0.70. The sample sizes aimed to be consistent with common psychometric cases in the literature with 500 and 1000 observations.

The simulation design allowed for a fully factorial design: 3 $\times$ 4 $\times$ 4 $\times$ 2 $\times$ 5 $\times$ 2 (number of factors $\times$ variables per factor $\times$ loadings $\times$ loading signs $\times$ correlations between factors $\times$ sample size) resulting in 960 full simulated condition combinations. For all conditions, 100 replicates were generated. All data were generated using the {latentFactoR} package (version 0.0.6; Christensen et al., 2024) in R (version 4.3.2; R Core Team, 2024).

### Data generation

Data generation followed the same approach as Golino et al. (2020). First, the population correlation matrix was generated,

$$ \mathbf {R_P} = \mathbf {\Lambda }\mathbf {\Phi }\mathbf {\Lambda ^\prime } + \mathbf {\Psi }, $$

where $\mathbf {\Lambda }$ is the factor loading matrix, $\mathbf {\Phi }$ is the matrix of correlations between factors, and $\mathbf {\Psi } = 1 - \text {diag}(\mathbf {\Lambda }\mathbf {\Phi }\mathbf {\Lambda ^\prime })$. Next, Cholesky decomposition was performed on the population correlation matrix such that:

$$ \mathbf {R_P} = \textbf{U}^\prime \textbf{U}. $$

If the population correlation matrix was not positive definite (i.e., at least one eigenvalue $\le$ 0) or any single item’s communality was greater than 0.90, then $\mathbf {\Lambda }$ was re-generated and the same procedure was followed until these criteria were met. The sample data matrix of continuous variables was computed:

$$ \textbf{X} = \textbf{ZU}, $$

where $\textbf{Z}$ is a matrix of multivariate normal data with rows equal to the sample size and columns equal to the number of variables.

### Loadings and correlations

#### Unrotated network loadings

All networks estimated in this study used the GLASSO with EBIC model selection following the approach applied by the EGA() function in the {EGAnet} package (version 2.0.7; Golino & Christensen, 2024). This approach differs from the commonly used {qgraph} package (Epskamp et al., 2012) in two ways. First, the default value of the lambda.min.ratio parameter, which sets the range of lambda values used in the grid search of network models that EBIC selects from, is set to 0.1 rather than {qgraph}’s default 0.01. This difference sets a higher lower bound on the lambda parameter which can lead to slightly sparser networks than {qgraph}’s EBICglasso() function on the same data. Second, a strategy is taken to mitigate the result of disconnected nodes in the network. If *any* nodes are disconnected in the network, the default value of 0.50 for the gamma parameter in EBIC, which controls sparsity, is decreased by 0.25 and the network is re-estimated with this lower gamma value. If the re-estimated network has *any* nodes that are disconnected, then gamma is set to zero and the network is re-estimated and the result, regardless of disconnected nodes, is retained. When gamma equals zero in EBIC, then the criterion is equal to the Bayesian information criterion.

Both of the original and revised network loadings were obtained using the net.loads() function in {EGAnet} (loading.method = "original" and loading.method = "revised", respectively). To obtain network loadings, a community membership for each node is necessary. To avoid the potential of inaccurate estimation of the number of factors or the variable placement in those factors, the simulated number of factors and variable assignments were provided.^3^ To compute network scores that would be used to estimate the correlations between factors, the loadings for both methods were pre-multiplied by the scaled data, $\textbf{OX}$. The correlations between these scores were used as the “unrotated” correlations between factors.

#### Rotation

All rotations in this study used the geominQ rotation (Browne, 2001), which was applied with ten random starts to avoid local minima using the {GPArotation} package (version 2024.3.1; Bernaards & Jennrich, 2005). Based on the number of factors, the epsilon parameter (eps) was adjusted to be 0.0001 for two factors, 0.001 for three factors, and 0.01 for four factors (Muthén & Muthén, 1998-2017). For the network loadings, the rotated loadings were used to compute scores in the exact same way as the unrotated loadings. The correlations between these scores were used as the “rotated” correlations between factors.

**Table 3:** Overall results

Parameter	Loading	Original		Revised		EFA
	Signs	Unrotated	Rotated	Unrotated	Rotated	
Loadings	All positive	0.940	0.954	0.953	0.966	0.973
	Half negative	0.432	0.437	0.952	0.965	0.974
Correlations	All positive	0.125	0.070	0.099	0.087	0.093
	Half negative	0.284	0.262	0.100	0.087	0.093

Note: All values are means within the respective method, rotation, and loading sign intersections. Values for loadings represent Tucker’s congruence coefficient (higher values are better); values for correlations represent mean absolute error (lower values are better)

#### EFA

As a benchmark, EFA loadings were estimated using the fa() function in the {psych} package (version 2.4.3; Revelle, 2024) using the aforementioned geominQ rotation. The number of simulated factors were supplied to the function. The model-implied correlations between factors (Phi) were used as the EFA correlations between factors.

#### Alignment

To ensure that all loadings and factor correlations were properly aligned for comparison with the simulated loadings and correlations between factors, the {fungible} package’s (version 2.4.4; Waller, 2024) faAlign() function was used.

### Statistical analysis

To evaluate the correspondence between the estimated and simulated loadings, Tucker’s congruence coefficient (also known as cosine; Tucker, 1951) was computed,

$$ \phi _T = \frac{\sum \textbf{xy}}{\sqrt{\sum \textbf{x}^2 \sum \textbf{y}^2}}, $$

where $x$ and $y$ represent vectors of the estimated and simulated loading matrices, respectively. The benefit of using Tucker’s congruence coefficient is that the similarity between loading matrices is estimated irrespective of differences in magnitude (Lorenzo-Seva & Ten Berge, 2006). Because network loadings are based on partial correlations, they are inherently smaller (usually) than zero-order correlations (van Bork et al., 2019). We followed Lorenzo-Seva and Ten Berge’s 2006 suggestion of fair similarity as $0.85<= \phi _T < 0.95$ and good similarity as $\ge 0.95$.

To evaluate the accuracy of the estimates of the correlations between factor, we used mean absolute error (MAE),

$$ MAE = \frac{\sum _{i=1}^n | \hat{r_{F_i}} - r_{F_i} |}{n}, $$

where $\hat{r_F}$ and $r_F$ are the estimated and simulated correlations between factors ($F$) and $n$ is the number of unique elements in $r_F$.

To evaluate the effects of the conditions, we focused solely on the non-varying conditions using analysis of variance (ANOVA). All ANOVAs were specified with each manipulated condition interacting with all other manipulated conditions. For the Varying conditions, the parameter that was varying (e.g., loading size) used the standard deviation of the replicate’s parameter. Using the standard deviation of the parameter allowed for effects related to parameter variability to be detected. Only main effects and interactions up to three parameters that reached at least a large partial eta-squared effect size ($\eta _p^2 \ge 0.14$; Cohen, 1988) are reported. Overall results for the varying conditions (variables, loadings, correlations between factors, and their interaction) are presented in a table.

## Results

### Overall

The overall congruence (cosine) and MAE were computed at the intersection of loading method, rotation, and loading signs (Table 3). There was a substantial difference in congruence for the original network loadings when all simulated loadings were positive (unrotated = 0.940 and rotated = 0.954) relative to when half of them were negative (unrotated = 0.432 and rotated = 0.437). This pattern was not evident in the unrotated (all positive = 0.953 and half negative = 0.952) and rotated (0.966 and 0.965, respectively) revised network loadings.

**Fig. 4:**
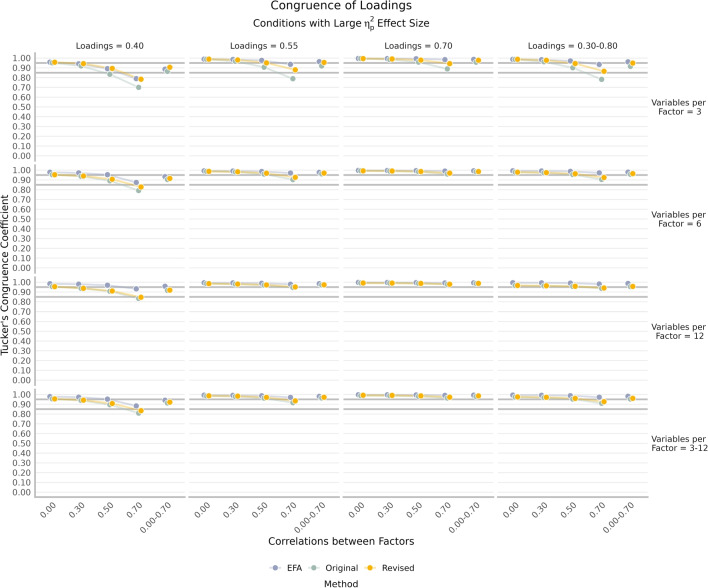
*Grey bars* on the congruence plot indicate fair (0.85) and good (0.95) congruence. The original and revised network loadings are unrotated; EFA loadings are rotated. Higher values are better

The MAE for the correlations between factors was similarly affected by signs where the original network loadings had substantially worse MAE when half the loadings had negative signs. This difference was not observed with the revised network loadings. When rotated, there were marginal increases in congruence and decreases in MAE for the network loadings, particularly for the revised network loadings (Table 1). EFA had the highest congruence but also highest MAE for factor correlations when all loadings were rotated.

Taken together, the overall results confirm a couple of expectations. First, the method to assign signs for the original network loadings was ineffective, leading to substantially worse congruence with the simulated loadings. Second, although congruence for the network loadings improved with rotation, the improvement was marginal. Similarly, the MAE of the factor correlations improved with rotation but the decreases were marginal. This result suggests that the (revised) network loadings can be used without rotation. Based on these overall findings, the rest of the results report the unrotated original and revised network loadings. Because the negative signs had such a substantial effect on the original network loadings, we moved forward with the positive loadings only. This decision allowed a more direct evaluation of the within-community sum (Eq. 3) and standardization (Eq. 4) revisions of the original network loadings.

### Loadings

There were several large, two-way interaction effects involving number of variables per factor, loadings, and correlations between factors (Fig. 4). All methods (EFA = $\eta _p^2 = 0.31$, original = $\eta _p^2 = 0.37$, revised = $\eta _p^2 = 0.33$) had a large interaction of loadings and correlations between factors such that as loadings decreased and correlations between factors increased their congruence decreased. The original ($\eta _p^2 = 0.40$) and revised ($\eta _p^2 = 0.15$) network loadings had a large interaction effect between number of variables per factor and correlations between factors such that as the number of variables per factor decreased and correlations between factors increased their congruence decreased. EFA had a large interaction effect between number of variables per factor and loadings such that as number of variables per factor and loadings decreased their congruence decreased. There was a large main effect of the number of factors such that congruence decreased as the number of factors increased for the original ($\eta _p^2 = 0.24$) and revised ($\eta _p^2 = 0.21$) network loadings.

**Table 4:** Overall congruence for varying condition results

Varying parameter	Original	Revised	EFA
Variables per factor (3–12)	0.947	0.954	0.976
Loadings (0.30–0.80)	0.939	0.954	0.982
Correlations between factors (0.00–0.70)	0.941	0.954	0.964
All three varying only	0.953	0.960	0.981

Note: All values are means within the respective method, rotation, and loading sign intersections. Values represent Tucker’s congruence coefficient between estimated and simulated loadings. The original and revised network loadings are unrotated; EFA loadings are rotated. Higher values are better

**Fig. 5:**
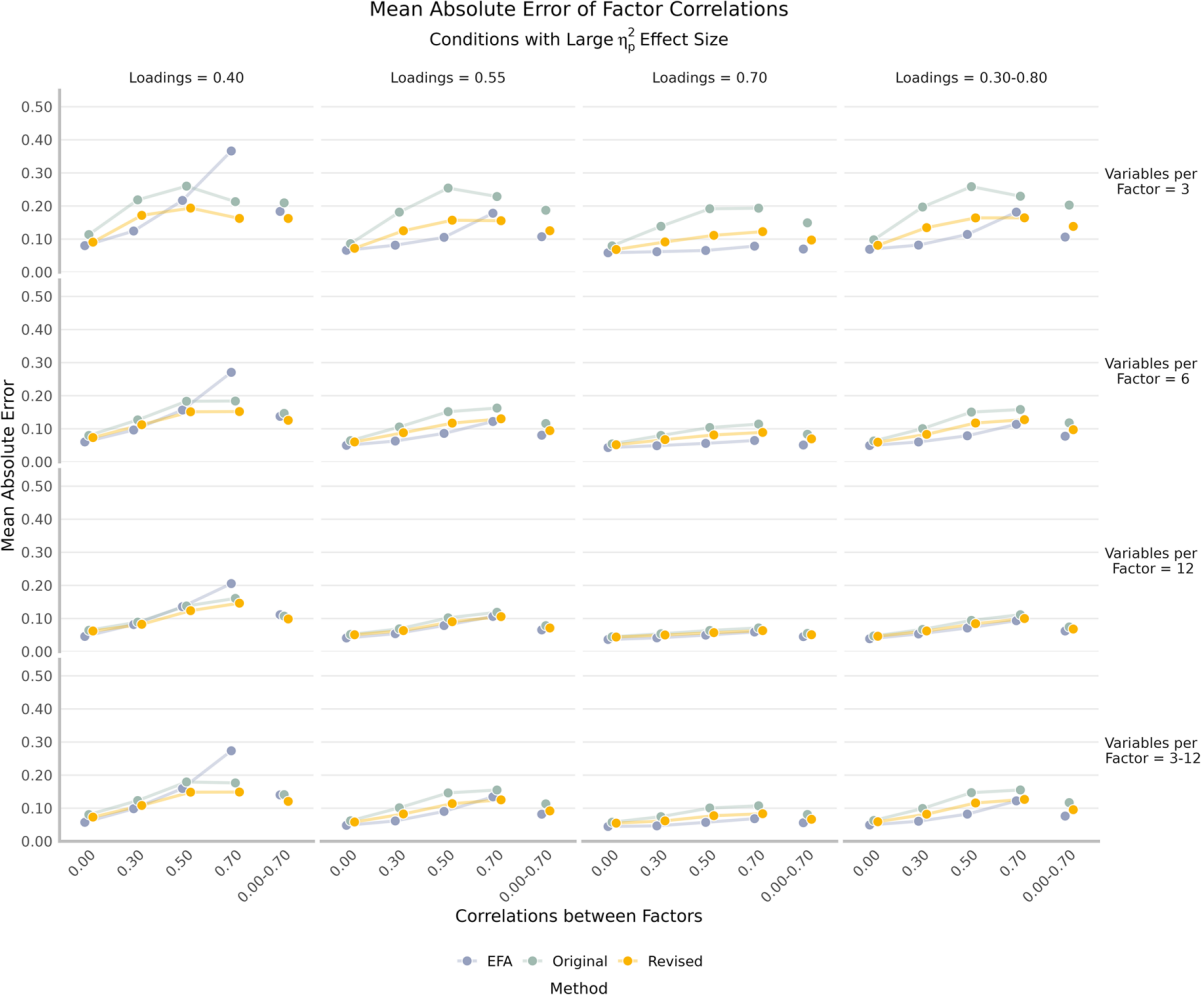
Mean absolute error for the correlations between factors. The original and revised network loadings are unrotated; EFA loadings are rotated. Lower values are better

The congruence for conditions where one or all three conditions (variables per factor, loadings, correlations between factors) were allowed to vary is reported in Table 4. Overall, variability in these parameters had marginal differences. The intersection, where all three conditions varied, showed the largest overall congruence relative to the other conditions. The revised network loadings were relatively more consistent in their congruence across the varying parameters relative to the original network loadings. Although the improvements of the revised network loadings over the original network loadings appear small, there is still improvement above and beyond fixing the signs. Based on Fig. 4, the majority of this improvement occurred when there were fewer variables per factor (3), reflecting the revisions demonstrated in Fig. 2. Overall, EFA loadings had higher congruence across all Varying conditions.

**Table 5:** Overall mean absolute error for varying condition results

Varying parameter	Original	Revised	EFA
Variables per factor (3–12)	0.114	0.095	0.095
Loadings (0.30–0.80)	0.131	0.102	0.084
Correlations between factors (0.00–0.70)	0.126	0.100	0.095
All three varying only	0.117	0.096	0.076

Note: All values are means within the respective method, rotation, and loading sign intersections. Values represent mean absolute error between estimated and simulated factor correlations. The original and revised network loadings are unrotated; EFA loadings are rotated. Lower values are better

### Factor correlations

There was one large, two-way interaction effect between loadings and correlations between factors for the MAE of the factor correlations for EFA ($\eta _p^2 = 0.35$). The effect was such that as loadings decreased and factor correlations increased the MAE increased. Low loadings (0.40) and very large correlations between factors (0.70) drove this effect, reaching MAE values (between 0.20 and 0.40) that were substantially larger than any of the other conditions (Fig. 5). These results should be interpreted with caution as they reflect conditions where there was also low loading congruence (Fig. 4).

All three methods (EFA $\eta _p^2 = 0.17$, original $\eta _p^2 = 0.33$, revised $\eta _p^2 = 0.14$) had a large main effect for the number of variables per factor such that as the number of variables per factor decreased the MAE increased. Both the original ($\eta _p^2 = 0.30$) and revised ($\eta _p^2 = 0.20$) network loadings had a large main effect of correlations between factors such that as the correlations between factors increased the MAE increased. The revised network loadings also had a large main effect for loadings ($\eta _p^2 = 0.16$) such that as loadings decreased the MAE increased.

For the conditions where one or all three parameters were varying, there was a clear improvement of the revised network loadings over the original network loadings (Table 5). In the cases of varying variables per factor and varying correlations between factors, the revised network loadings MAE, on average, was on par with EFA.

### Mapping effect size guidelines

Because network loadings use (regularized) partial correlations, their magnitudes are (usually) much smaller than their factor loading and zero-order counterparts (van Bork et al., 2019). To establish meaningful measures of loading effect size guidelines, such as those provided in factor analysis (i.e., 0.40, 0.55, 0.70; Comrey & Lee, 2013), an additional simulation was performed. For this simulation, the goal was to cover a broad factor analytic parameter space. Following the same data generation procedure as the simulation above, data were generated from two, three, four, and five factors with three, six, nine, twelve, and twenty-four variables per factor. Loadings and correlations between factors were generated in ranges of 0.30–0.80 and 0.00–0.50, respectively. The rationale for generating these ranges was that the congruence with the simulated factor loadings were most variable under these conditions. Five hundred replicates for each of the twenty conditions were generated.

**Fig. 6:**
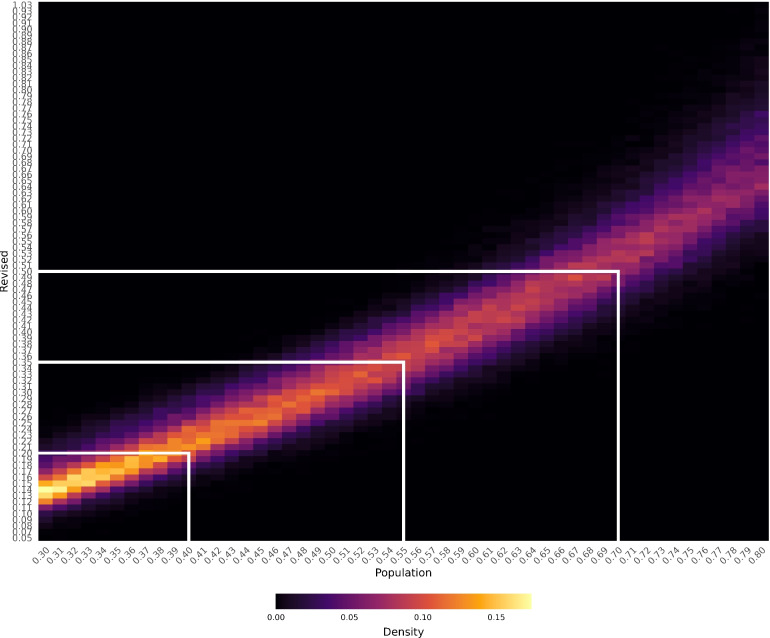
Binned loadings heatmap where the density of the bins increases from darker (*black*) to lighter (*yellow*) colors

The simulated loadings were regressed on the revised network loadings using a linear model to obtain the $R^2 = 0.905$. Tucker’s congruence coefficient was also computed between the two, finding $\phi _T = 0.981$. The larger $\phi _T$, relative to Table 3, is likely due to the exclusion of large correlations between factors (0.70), which was the most detrimental condition for congruence between the simulated and revised network loadings.

Figure 6 depicts a heatmap where the simulated and revised network loadings were binned by rounding their respective values to two digits. The heatmap provides an indication of where the lowest to greatest density of values (from darker to lighter) are for the revised network loadings with respect to the corresponding simulated loading size. White bars were added to visually center where the revised network loadings align most with the simulated population loading sizes of 0.40, 0.55, and 0.70. Based on the heatmap, these values roughly corresponded with revised network loadings of 0.20, 0.35, and 0.50. The densities get weaker toward the higher end of the simulated loading spectrum, so the revised network loading of 0.50 is slightly lower than the true center mass of the simulated population loading of 0.70. Nonetheless, these guidelines provide rough effect sizes of small (0.20), moderate (0.35), and large (0.50) revised network loadings.

Importantly, loadings lower than 0.30 were excluded from these plots. This exclusion was intentional as values lower than 0.30 were not specifically generated and therefore would reflect cross-loadings. Due to the nature of the revised network loadings based on regularization in networks, the correspondence between small factor loading values (e.g., $< 0.30$) and network loadings is expected to be less prominent. Furthermore, for psychometric purposes, factor loadings $\ge 0.40$ are usually considered for main loadings and $\ge 0.25$ for cross-loadings. Therefore, the mapping between the revised network loadings and simulated population loadings captures the most common range of interest for psychometric applications.

## Discussion

Loadings are a fundamental tool for psychometric measurement in latent variable models. This study added several key revisions to the original network loadings put forward by Christensen & Golino (2021) including more precise sign determination, mitigation of larger cross-loadings relative to assigned loadings, and independence of loading size from the number of variables per factor. These adjustments provided marked improvements to the congruence of network loadings with the simulated factor loadings. The simulation also extended the original simulation to evaluate the correlations between factors, finding that the network loadings are best left unrotated. Overall, the revised network loadings provide an improved heuristic to quantify factor loadings when the data generating model is a factor model. Importantly, the EFA factor loadings had the most accurate estimates, on average, of the simulated parameters and should remain the preferred method when data are believed to be generated from a factor model.

Although the EFA factor loadings performed as well as or better than the revised network loadings across conditions, there are a couple of reasons why researchers might prefer network loadings over factor loadings. First, if the data generating model is something other than a factor model, then it’s possible that network loadings could extend to these structures especially when assumptions of a factor model might be violated (Ouyang et al., 2023). Additional research is necessary to explore this possibility as well as how network loadings should be interpreted and evaluated in these contexts. Second, the revised network loadings do not require rotation to provide a simple structure (when correlations between factors are low) or to obtain correlations between factors. Factor rotations are a notorious source of variability in factor analytic applications (Sass & Schmitt, 2010), so the trade-off of one less researcher degree of freedom for slightly less congruent loadings and precise correlations between factors may be worthwhile to some.

Across the simulation conditions, the revised network loadings achieved, at minimum, fair congruence ($\phi _T \ge 0.85$) except for the condition where loadings were small (0.40), correlations between factors were very large (0.70), and there were few variables per factor (3). In this same condition, the rotated EFA loadings struggled comparably. The lower congruence in this condition is not surprising given that lower loadings lead to sparser networks while larger correlations between factors lead to increased between-community connections. Nevertheless, the revised network loadings were consistently on par or just a step below the EFA loadings across all conditions, suggesting that they capture roughly the same patterns in the data.

When signs were considered, the original network loadings did not achieve fair congruence in any conditions (see Table 3). Although the congruence for the original network loadings was much better when loadings were all positive, the number of variables per factor is still a likely issue (e.g., few variables per factor). This simulation, like the previous one conducted by Christensen and Golino (2021), did not include variables per factor above twelve. Therefore, the pronounced effects observed in the Introduction example (see Fig. 2) were reduced due to the simulated conditions (e.g., restricted variability in the difference in the number of variables per factor), suggesting that the current congruence observed in the simulation is likely optimistic. Nevertheless, the revised network loadings still demonstrated some improvement over the original network loadings across conditions.

A novel contribution of this study is the evaluation of the correlations between factors. The network scores, computed similarly to component scores (i.e., loadings pre-multiplied by data), fared well and were within 0.10, on average, of the simulated correlations across all conditions. Notably, although loading congruence improved with rotation, the correlations between factors improved marginally. This result is significant as rotation is a common and necessary part of loading and score estimation in factor models. Our findings indicate that the revised network loadings can be left unrotated without much loss in accurate parameter estimation.

The importance of the revised network loadings permeates throughout psychometrics with networks. From invariance (Jamison et al., 2024) to hierarchical dimensionality (Jiménez et al., 2023), the revised network loadings proposed here should increase the accuracy and validity of measurement quality assessed by network models when the data generating model is a factor model. The results of this simulation are particularly relevant for hierarchical dimensionality assessment. Hierarchical structures using network models rely on the accuracy of the correlations between factors when estimating each order of the hierarchy. This accuracy is even more relevant with the more lower-order factors there are. One recent example comes from the personality literature where the 300-item NEO-IPIP was assessed using hierarchical EGA (Samo et al., 2023). In this study, the authors found 30 lower order factors that ranged in size from 3–24 variables. Although this dataset would have spelled trouble for the original network loadings, the revised network loadings can appropriately handle the presence of positive and negative signs on the same factor (Table 3), diversity in the number of variables per factor (Fig. 2), and the (likely) variability in the size of the correlations between factors. In addition, the revised network loadings are expected to align with the communities and items placed in those communities by the community detection algorithm whereas estimating the number of factors and factor loadings with EFA based on the EGA number of factors would likely lead to different results.^4^

The revised network loadings also have implications for researchers interested in evaluating network theory (Borsboom, 2017). Comorbidity is intimately tied to the network perspective of psychopathological disorders (e.g., Cramer et al., 2010) with researchers proposing measures such as stabilizing (within-community connections) and communicating (between-community connections) symptoms (Blanken et al., 2018) with the latter being extended to bridge centrality (Jones et al., 2021) which purportedly quantifies the extent to which a symptom is a pathway to comorbidity (Christensen et al., 2021). Researchers often seek to differentiate symptoms that are “bridges” from symptoms that are overlapping or comorbid (Santiago et al., 2024). Although bridging and overlapping symptoms are viewed as phenomenologically different in theory (Jones et al., 2021), its not clear from existing network measures how this distinction can be made in practice and subsequently whether the distinction itself is clinically meaningful. Importantly, this study identified key contributions to between-community connections, when data are generated from a factor model, beyond cross-loadings: correlations between factors (Fig. 3). Between-community connections likely reflect a combination of both cross-loadings and correlations between factors, offering some insight into this distinction. Comorbidity might be defined as correlations between factors since the clinical view is that two pathologies co-occur whereas bridge symptoms might be defined as large cross-loadings for individual symptoms to other factors (Christensen et al., 2021). Since both are likely to contribute to between-community connections, network loadings may offer a way to disentangle these effects by modeling cross-loadings and correlations between communities. In any case, the nature of this distinction is unlikely to be categorical or mutually exclusive and the (revised) network loadings afford the potential for a spectrum between the two to be examined (Christensen et al., 2021).

Despite the substantive implications of between-community connections, their combination of cross-loadings and correlations between factors can be problematic from a factor analytic perspective. Disentangling these effects could be as simple as identifying whether all variables in a community have (roughly) equivalent size cross-loadings, leading toward an interpretation of correlation between factors or a few variables have substantially larger cross-loadings relative to all others, leading toward an interpretation of cross-loadings. This simple breakdown, however, is unlikely to match reality. Future simulation studies should investigate the extent to which substantial cross-loadings versus correlations between factors contribute to network cross-loadings.

A primary limitation of this study is that data were generated from a factor model and the adjustments made to the revised network loadings were based on the assumption of a (multidimensional) factor model. This limitation raises several questions regarding how network loadings should be interpreted when the data generating model is a network model and how they fit within a network theory of measurement (van Bork et al., 2024). To position network loadings within a network theory of measurement, we start with the premise that the connections between variables are direct, mutual reinforcing relations (Borsboom, 2017). Communities that form from these relations are emergent such that they appear because of direct relations between variables rather than a single latent cause (Cramer et al., 2012). Network loadings then correspond to each node’s contribution to the emergence of a community in the network (Christensen & Golino, 2021; Christensen et al., 2020). This interpretation suggests that network scores, as computed in this study, represent summary statistics for the current state of each community (Cramer, 2012; van Bork et al., 2024).

Measurement quality will also depend on the measurement theory. Within the latent variable framework, factor loadings quantify the extent to which each indicator measures the latent factor. Measurement quality in network theory places greater emphasis on variable selection (Bringmann et al., 2022; Henry & Ye, 2024; Neal & Neal, 2023). With networks, the boundaries of network communities can be fuzzy, allowing the potential for variables to overlap (Blanken et al., 2018; Lange, 2021; Schmittmann et al., 2013). To determine the (fuzzy) boundaries of communities and possible overlapping communities, a decision point is crucial, and network loadings could be one metric to establish these boundaries (Santiago et al., 2024). With an emphasis on variable selection, a low network loading may not be as problematic as it would be for a factor model such that the variable can contribute to the emergence of the network structure (and therefore overall system) but does not contribute strongly to any single dimension in the network. The variable may then contribute to the system as a whole but not to the emergence of a particular community in the network. In other circumstances, roughly equivalent network cross-loadings might suggest a fuzzy boundary between two (or more) network dimensions (Santiago et al., 2024), and loadings that are larger for a different community than the theoretical community might suggest that a variable is better placed in that community.

Another limitation of the simulation is that all data were generated as continuous. This choice was intentional as continuous data provided the most ideal conditions in which to compare the improvements of the revised network loadings over the original network loadings but future work should determine whether polytomous or dichotomous data have substantial effects especially considering that many psychometrics networks rely on ordinal (e.g., personality) and binary (e.g., psychopathology) variables. Based on the original simulation for the original network loadings, which found that there was no substantial difference between continuous and polytomous data, the expectation is that there would be little to no effect (Christensen & Golino, 2021).

In sum, this study provides several revisions to the original network loading formulation and demonstrates that these revisions make them more robust. These loadings form a foundation on which network psychometrics might be able to extend to other psychometric procedures. Because the revised network loadings adjust for the communities and number of nodes in those communities, they mitigate many of the latent confounds (e.g., community structure, number of nodes in the network) that affect other centrality measures such as strength and expected influence (Hallquist et al., 2021). Therefore, the revised network loadings provide a standardized metric that mitigates most latent confounds in psychometric networks (when data are generated from latent factors), providing a more direct and valid approach to understand each node’s contribution to the emergence of each community and the network structure.

This study paves the way for further research in network psychometrics that relies on the computation of network loadings. The findings from our simulation study suggest that these revised network loadings can be leveraged to create innovative psychometric methods using network approaches, paralleling the development of traditional psychometric techniques. This development has started in recent years, from the development of metric invariance methods for exploratory graph analysis (Jamison et al., 2024) to hierarchical exploratory graph analysis (Jiménez et al., 2023). With these revised network loadings, we anticipate the emergence of additional psychometrics methods such as those found in item response theory (Muraki & Carlson, 1995).

## Supplementary Information

Below is the link to the electronic supplementary material.

Supplementary file 1 (pdf 30 KB)

## Data Availability

All data can be found on the Open Science Framework.

## Footnotes

1 We use factor analysis terminology throughout the manuscript because all data generated in the simulation are from factor models. Different terminology, such as “communities,” from the network literature do not necessarily imply a latent factor. For consistency and brevity, we use “factor” for both factor and network models. If data were not generated from factor models, then terminology can be converted to the appropriate terms such as “correlations between communities” rather than “correlations between factors.” We later discuss, however, that the interpretation of communities, loadings, and scores may change when data are generated from a network model.

2 This general form, $\frac{1}{\log {(p)}}$, tends to hold when zero-order correlations are not all the same. When the range of zero-order correlations is more extreme and variable, there tends to be a less pronounced decrease in the partial correlations as the number of variables increases. A small scale simulation was implemented to demonstrate this effect using a unidimensional factor model with loadings that ranged from 0.10–0.90 and the number of variables that ranged from 2–100. In short, there was an *upward* bias such that the partial correlations did not decrease as rapidly evident in Fig. 1. This simulation and result are available on our Open Science Framework page.

3 To put network loadings on equivalent footing as the EFA loadings, a modified version of the Walktrap algorithm (Pons & Latapy, 2006) was used to assign item placements based on the number of simulated factors (rather than assigning items to the simulated item placements). Although there was little difference overall, misspecification of the item placements did lead to lower congruence and higher MAE for the network loadings. These results are reported in the Supplemental Information.

4 Using the authors’ data from their Open Science Framework project page, we confirmed that the largest absolute revised network loading achieves a normalized mutual information (NMI; Danon et al., 2005) of 1 (i.e., exact same item placement) with the item placement of the community detection algorithm. In contrast, the original network loadings achieves an NMI of 0.944 and EFA estimated with 30 factors and geominQ rotation achieves 0.823 with the item placement of the community detection algorithm.

## Code Availability

All R scripts can be found on the Open Science Framework.
