# Supplementary material for: Revised network loadings
Source: Behav Res Methods. 2025 Mar 14;57(4):114. doi: 10.3758/s13428-025-02640-3 (PMC11909041; doi:10.3758/s13428-025-02640-3)
Supplement: Supplementary file 1 — (pdf 30 KB) [file 13428_2025_2640_MOESM1_ESM.pdf]

Supplementary Information

Revised Network Loadings

SI 1. Revised Network Loadings with Specified Number of Factors

The main text simulation used the simulated item placements for the revised network loadings whereas the simulated number of factors were provided for EFA. In order to put the revised network loadings on an even footing and to explore the effects of item placement misspecification, two additional comparisons were added. First, a modified version of the Walktrap algorithm (Pons & Latapy, 2006) using {igraph} (version 2.0.3; Csardi & Nepusz, 2006) was applied to estimated network. This modified version extracted the dendrogram estimated by the Walktrap algorithm and made a "cut" to arrive at the same number of clusters as the simulated number of factors. This approach differs from the standard Walktrap algorithm which uses modularity (Newman, 2006) to determine where the optimal cut should be made (based on the highest modularity value). The revised network loadings were then estimated, drawing a more direct comparison to EFA. To distinguish the two versions of the revised network loadings the simulated item placements were called "Revised with Item Placements" and the simulated number of factors were called "Revised without Item Placements."

To determine the extent of item placement misspecification, the adjusted Rand index (ARI; Hubert & Arabie, 1985) was computed between the simulated item placements and the item placements of the modified Walktrap algorithm. ARI computes a similarity between the two sets of item placements ranging from -1 (completely dissimilar) to 1 (identical) with 0 corresponding to no better than random. It was expected that as the level of item placement misspecification increased (i.e., lower ARI) the congruence with the simulated loadings would decrease and MAE with the simulated factor correlations would increase.

Table 1
Misspecification Results

Parameter	Condition Set	Revised with Item Placements	Revised without Item Placements	EFA
Loadings	All	0.956	0.951	0.975
	Misspecified	0.870	0.828	0.900
Correlations	All	0.099	0.099	0.071
	Misspecified	0.156	0.156	0.112

Note: All revised network loadings used unrotated loadings; EFA used rotated loadings. All values are means within the respective method compared to the population values except for Revised Comparison which compares Revised with Item Placement to Revised with Factors. Values for loadings represent Tucker's Congruence Coefficient (higher values are better); values for correlations represent mean absolute error (lower values are better).

In total, 10.4% of conditions resulted in at least one misspecified item placement. To achieve more precise congruence and MAE estimates for the misspecified cases, the simulation was performed with 1000 replicates (relative to 100 in the main text simulation). The results across all conditions (All) and conditions with at least one item placement misspecified (Misspecified) are presented in Table 1. Overall, there was only a slight difference in the Revised with Item Placement and Revised without Item Placements congruence and MAE. For the conditions where there was at least one misspecified item placement, there was a difference, on average, around 0.042 relative to the Revised with Item Placements with the simulated loadings, but no difference, on average, for their MAE with the simulated factor correlations. The correlation between the level of item placement misspecification and congruence for the Revised Comparison was strongly correlated, $r(99965) = 0.57, p < 0.001$, and moderately correlated with MAE, $r(99965) = -0.31, p < 0.001$. These results suggest that as the amount of item placement misspecification increases, the congruence and MAE with the correct item placements decreases and increases, respectively. It's important to point out: The congruence and MAE for the EFA loadings were also substantially lower and higher (respectively) overall in the Misspecified conditions suggesting that these conditions are particularly challenging to recover the simulated parameters.
